# Covert hepatic encephalopathy as a multi-organ syndrome: the gut–liver–muscle–brain axis, diagnosis, treatment, and multidisciplinary care

**DOI:** 10.1007/s00535-026-02425-1

**Published:** 2026-05-08

**Authors:** Takao Miwa, Cynthia L. Hsu, Masahito Shimizu, Patricia P. Bloom, Bernd Schnabl

**Affiliations:** 1 Department of Medicine, University of California San Diego, 9500 Gilman Drive, La Jolla, CA 92093 USA; 2 Department of Gastroenterology/Internal Medicine, Graduate School of Medicine, Gifu University, 1-1 Yanagido, Gifu, 501-1194 Japan; 3 Department of Medicine, VA San Diego Healthcare System, 3350 La Jolla Village Drive, San Diego, CA 92161 USA; 4 Department of Medicine, Mayo Clinic, 200 First Street SW, Rochester, MN 55905 USA

**Keywords:** Frailty, Malnutrition, Microbiome, Minimal hepatic encephalopathy, Sarcopenia

## Abstract

Covert hepatic encephalopathy (CHE) is a highly prevalent complication of liver cirrhosis. Despite the absence of overt symptoms, CHE is strongly associated with impaired quality-of-life, overt hepatic encephalopathy, and mortality. Over the past two decades, evidence regarding the pathophysiology, diagnosis, and treatment of CHE has accumulated considerably, and clinical guidelines recommend screening in patients with cirrhosis. Nevertheless, diagnostic and therapeutic algorithms have not been fully implemented in real-world practice, and many patients remain undiagnosed and untreated. Understanding the natural history of CHE is essential to improve cirrhosis care, as it provides a framework for appropriate screening, treatment decision-making, and patient counseling. CHE is a multi-organ syndrome with complex interactions between the liver, gut, skeletal muscle, kidneys, and brain, with impaired ammonia handling and systemic inflammation acting as central drivers of this organ crosstalk. Hyperammonemia induces astrocytic dysfunction, brain edema, and neuroinflammation, while systemic inflammation, oxidative stress, sarcopenia, gut dysbiosis, and altered microbial metabolites, including bile acids and short-chain fatty acids, further modulate disease expression. In this review, we summarize current understanding of CHE pathophysiology, diagnostic testing, including psychometric batteries and point-of-care tools, such as the Stroop test and animal naming test, and therapeutic options, ranging from lactulose and rifaximin to microbiome-targeted approaches, including fecal microbiota transplantation. We also highlight major challenges in CHE management, including limited implementation of testing, inadequate biomarkers, diagnostic difficulties in geriatric cirrhosis, and unmet needs in fall and driving risk management, and emphasize the importance of multidisciplinary team-based approaches to improve patient outcomes.

## Introduction

Cirrhosis is the late stage of chronic liver disease, characterized by advanced fibrosis, disruption of normal liver architecture, and progressive loss of liver function [1]. Although the underlying etiology of cirrhosis has shifted from viral hepatitis to steatotic liver disease, progression to cirrhosis is inevitably accompanied by clinical complications. Hepatic encephalopathy (HE), which is one of the most debilitating complications of cirrhosis, includes a wide spectrum of cognitive decline, ranging from subclinical alterations to coma [2–6]. HE is broadly classified into overt HE (OHE), with clinically apparent neurological signs such as disorientation or asterixis, and covert HE (CHE), including minimal HE (MHE) and West Haven grade I **(**Table 1**)** [2–6]. CHE is defined by the presence of neuropsychological/neurophysiological abnormalities, such as deficits in attention, working memory, response inhibition, and visuomotor coordination, in the absence of overt neurological signs, and is detectable only through specialized testing [2–6]. The term CHE was retained to avoid confusion from repeated nomenclature changes, to define OHE as West Haven grade ≥ II for clinical consistency, and because the distinction between MHE and grade I HE is difficult, and much of the existing literature applies to both [4]. CHE is recognized as an important issue in cirrhosis care [7, 8], and global liver societies recommend routine screening in this population [2–6]. However, many patients remain undiagnosed and untreated, largely due to challenges in testing and treatment [7, 8].

**Table 1:** Classification and features of HE

ISHEN criteria [3, 4]	West Haven criteria [2]	Clinical features	Detectability
Covert HE	Minimal HE	No overt mental status changes; impaired psychomotor speed, executive function, attention, or visuomotor coordination	Detectable only by specialized psychometric or neurophysiological testing
	Grade I	Trivial lack of awareness, shortened attention span, impaired calculation, altered sleep rhythm, mild euphoria or anxiety	Subtle abnormalities on clinical examination; require targeted testing for confirmation
Overt HE	Grade II	Lethargy or apathy, disorientation for time, personality change, inappropriate behavior, dyspraxia, asterixis	Detectable on routine clinical examination
	Grade III	Somnolence to stupor, confusion, gross disorientation, bizarre behavior; responsive to stimuli	Easily detectable on clinical examination
	Grade IV	Coma; unresponsive even to painful stimuli	Clinically evident coma

*HE* hepatic encephalopathy, *ISHEN* International society for hepatic encephalopathy and nitrogen metabolism

Accumulating evidence indicates that CHE should not be regarded as an isolated cerebral disorder, but rather as a multi-organ syndrome arising from complex interactions among the liver, gut, skeletal muscle, and brain [9, 10]. In particular, dysregulation of the gut-liver-muscle-brain axis, driven by hyperammonemia, systemic inflammation, sarcopenia, altered bile acid signaling, and gut dysbiosis, plays an important role in the pathogenesis and progression of CHE [9, 10]. Given its impact on outcomes, effective CHE management requires an integrated understanding of its multi-organ pathophysiology together with practical diagnostics and evidence-based therapies. In this context, ammonia-lowering agents target impaired nitrogen metabolism, nutritional optimization and branched-chain amino acid (BCAA) supplementation address sarcopenia and metabolic imbalance, and microbiome-directed therapies aim to correct dysbiosis, which contributes to CHE pathogenesis. This review summarizes CHE as a multi-organ syndrome, focusing on the gut-liver-muscle-brain axis, diagnosis, treatment, and future challenges and directions for clinical implementation.

## Natural history, and clinical relevance of CHE

The global prevalence of CHE in patients with cirrhosis is estimated at 40.9%, with rates of 48.4% in South-East Asia, 38.2% in the Americas, 37.2% in Europe, and 36.5% in Western Pacific regions [11]. The prevalence rises in parallel with worsening liver functional reserve, increasing from 29.3% in Child–Pugh class A to 45.9% in class B and 61.6% in class C [11]. CHE is known to impair quality-of-life through sleep disturbances and subtle neurocognitive dysfunction [12, 13]. In addition, CHE slows responses to external stimuli and is an important risk factor for critical daily events, such as motor vehicle accidents and falls [14–19]. The most important clinical outcome of CHE is its progression to OHE with an annual incidence rate of 10% [20]. Patients with CHE have an approximately two- to five-fold higher hazard of developing OHE compared with those without CHE [21, 22]. Furthermore, since CHE is an early sign of decompensation, the hazard ratio for mortality in patients with CHE is two to four times higher in affected patients [23, 24]. This high prevalence and its impact on clinical outcomes clearly highlight the importance of CHE in the management of cirrhosis.

## Pathophysiology of CHE

### Central role of ammonia in CHE

**Fig. 1:**
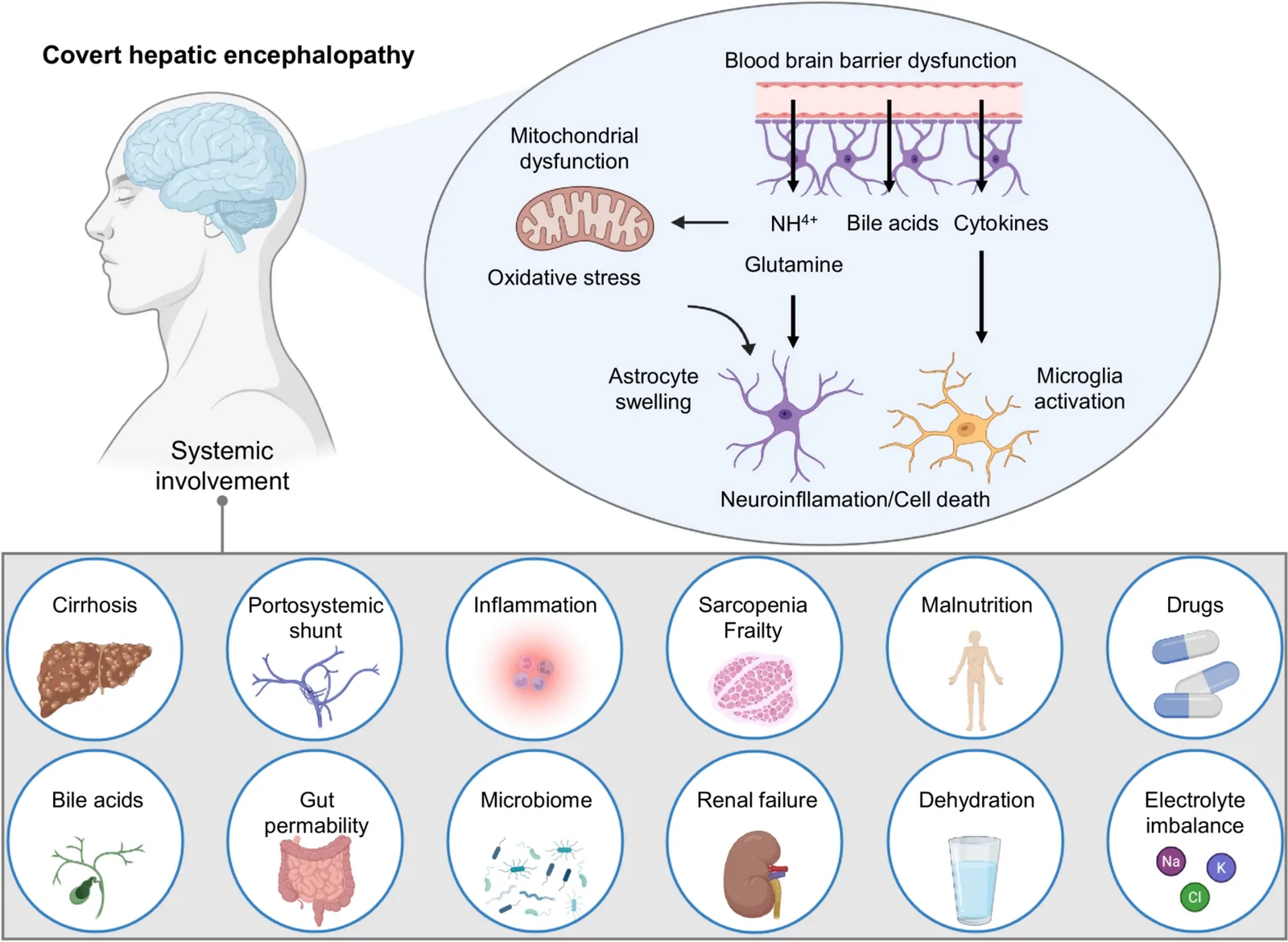
Multi-organ and multifactorial pathophysiology of covert hepatic encephalopathy. In the brain, ammonia, bile acids, and inflammatory cytokines cross the blood–brain barrier, inducing glutamine accumulation, mitochondrial dysfunction, and oxidative stress in astrocytes, leading to astrocyte swelling, microglial activation, neuroinflammation, and ultimately cell death. Cirrhosis and portosystemic shunts drive hyperammonemia. Systemic inflammation synergizes with ammonia, while sarcopenia and frailty reduce skeletal muscle-mediated ammonia detoxification. Malnutrition increases cerebral vulnerability through impaired energy and nutrient supply. Alterations in the gut-liver axis, including increased gut permeability, microbiome dysbiosis, and altered bile acid signaling, modulate ammonia handling and brain function. Renal failure, dehydration, electrolyte imbalance, and drugs further impair ammonia clearance and precipitate covert hepatic encephalopathy

CHE pathophysiology is centered on impaired liver functional reserve and neurocognitive dysfunction arising from brain alterations, but also involves complex multi-organ interactions, in which hyperammonemia, inflammation, sarcopenia, and the gut–liver axis play critical roles **(**Fig. 1**)**. In patients with HE, increased blood–brain barrier permeability facilitates the translocation of multiple neuroactive substances, such as ammonia, bile acids, and inflammatory cytokines [25]. Among them, ammonia plays a central role in the pathogenesis of CHE. In the brain, ammonia is detoxified to glutamine by glutamine synthetase in astrocytes. Cerebrospinal fluid glutamine levels are elevated in HE and correlate with disease severity, supporting a role for ammonia-related metabolism in its pathogenesis [26, 27]. In addition, ammonia impairs mitochondrial energy metabolism by inhibiting key enzymes of the tricarboxylic acid cycle, such as α-ketoglutarate dehydrogenase, leading to ATP depletion [25]. Furthermore, ammonia and ammonia-derived glutamine induce the mitochondrial permeability transition and promote oxidative and nitrosative stress, thereby amplifying astrocyte dysfunction [28]. Ammonia further disrupts astrocyte ion and water homeostasis through activation of multiple transporters and channels and ultimately leads to astrocyte swelling [29, 30]. Beyond direct astrocytic toxicity, ammonia interacts with systemic inflammation to promote microglial activation and subsequent neuroinflammation [31]. In addition, experimental models of HE suggest that ammonia-related neurotoxicity may extend beyond astrocytic dysfunction to impaired hippocampal synaptic plasticity [32]. Together, these mechanisms form a vicious cycle driving HE, with impaired ammonia metabolism as one of the best-studied and most important features of CHE pathophysiology.

### Ammonia metabolism in the liver, intestine, muscle, and kidney

Ammonia is mainly generated in the intestine through the breakdown of glutamine, urea, and nucleotides, and its production is further enhanced by ammonia-producing bacteria, such as *Clostridium* spp., *Enterococcus* spp., *Escherichia coli*, and *Shigella* spp. [9, 33]. In the liver, glutaminase 2 generates ammonia from glutamine for urea synthesis, whereas glutamine synthetase scavenges excess ammonia by converting glutamate to glutamine in an ATP-dependent process [9]. Hyperammonemia develops when ammonia production surpasses its elimination through the urea cycle, which relies on five key enzymes including carbamoyl phosphate synthetase 1, ornithine transcarbamylase, argininosuccinate synthase, argininosuccinate lyase, and arginase 1 [34]. In patients with cirrhosis, liver functional reserves are markedly reduced, and the capacity of the urea cycle to detoxify ammonia is impaired. Zinc deficiency impairs ornithine transcarbamylase activity and further decreases urea cycle function [20, 35]. Since the liver is the central organ of ammonia detoxification, portosystemic shunts allow portal blood to bypass the liver and cause hyperammonemia and HE. In fact, hyperammonemia resulting from portosystemic shunts improves after shunt occlusion [36]. Ammonia-lowering therapy restored urea-cycle function and significantly reduced cell death, inflammation, and fibrosis in a rat model of metabolic dysfunction-associated steatotic liver disease, indicating its potential in liver disease [37].

Skeletal muscle serves as an important metabolic organ for ammonia production and detoxification. Ammonia in muscle is generated mainly through ATP degradation and amino acid metabolism, and detoxified via glutamine synthetase using BCAAs [38, 39]. BCAAs provide the primary nitrogen source for glutamate synthesis, which in turn enables ammonia detoxification through glutamine synthetase [40]. Glutamine is released into the circulation in exchange for BCAA uptake, thereby linking ammonia handling with BCAA metabolism [40]. While skeletal muscle detoxifies ammonia, hyperammonemia decreases serum BCAA and increases myostatin levels, both of which result in inhibition of mammalian target of rapamycin signaling and decreased protein synthesis [41, 42]. In addition, hyperammonemia activates the general control non-derepressible 2 which inactivates the eukaryotic initiation factor 2 and impairs protein synthesis [41, 43]. Consequently, sarcopenia, characterized by loss of muscle mass and strength, is a robust biomarker for CHE and OHE [44–46]. Furthermore, ammonia-lowering therapy is a potential treatment not only for HE, but also for sarcopenia, with benefits for both muscle mass and strength [47, 48]. These findings underscore the pivotal role of skeletal muscle in linking BCAA metabolism with ammonia detoxification, which has important implications for both HE and sarcopenia in cirrhosis.

**Fig. 2:**
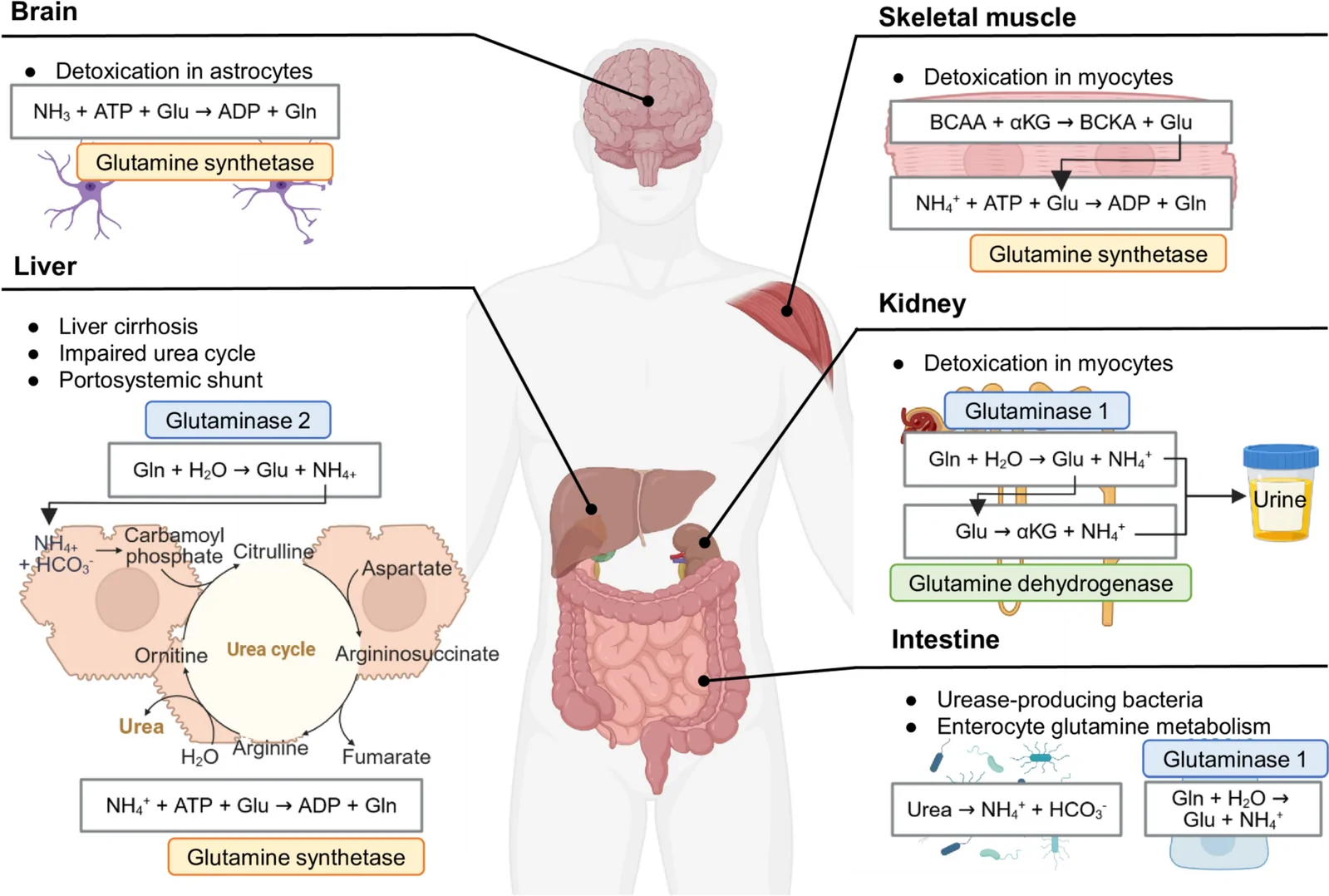
Multi-organ involvement in ammonia metabolism. Ammonia is primarily generated in the intestine by urease-producing bacteria, which hydrolyze urea to ammonia, and by glutamine catabolism mediated by glutaminase. Under physiological conditions, ammonia detoxification occurs mainly through the urea cycle with additional perivenous scavenging via glutamine synthetase. In patients with cirrhosis, impaired urea cycle activity and portosystemic shunting markedly reduce hepatic ammonia clearance. Extrahepatic organs, including the brain and skeletal muscle, compensate by incorporating ammonia into glutamine via glutamine synthetase, at the cost of increased adenosine triphosphate (ATP) consumption. In skeletal muscle, this process is closely coupled with increased utilization of branched-chain amino acids, which serve as important nitrogen donors and support glutamate and glutamine synthesis. Simultaneously, enhanced glutaminase activity in the intestine and kidney contributes to systemic ammonia production, while renal glutamate dehydrogenase modulates ammonia generation and excretion. *ATP* adenosine triphosphate, *ADP* adenosine diphosphate, *BCAA* branched-chain amino acid, *BCKA* branched-chain keto acids, *Glu*, glutamate, *Gln* glutamine, *α-KG* alpha-ketoglutarate

The kidneys are also an important organ in ammonia metabolism. The kidneys contribute to ammonia homeostasis by producing ammonia through glutaminase activity, resulting in stable ammonia levels and acid–base balance [49]. In proximal tubule cells, glutamine is converted to ammonia by the action of glutaminase 1 and glutamate dehydrogenase in the mitochondria and secreted into the tubular lumen [50]. The hyperdynamic circulation in cirrhosis activates the renin-angiotensin system and other vasoconstrictor hormones, which then stimulate glutaminase 1 activity; as a result, renal ammonia production contributes to systemic hyperammonemia [49]. Ammonia is excreted into the urine after reabsorption of ammonium in the thick ascending limb cells and secretion into the urine in the collecting duct [50]. Renal ammonia handling is driven primarily by acid–base disturbances and liver failure, with the kidneys promoting ammonia excretion to limit systemic accumulation in advanced liver disease or after portosystemic shunting [51]. Indeed, approximately 70% of renal ammonia is released into the systemic circulation in healthy rats, while 30% is released into the systemic circulation and 70% is excreted in the urine in rats with liver failure [52]. Clinically, the importance of renal regulation is demonstrated by the observation that dehydration caused by diuretics can trigger HE, which improves with fluid resuscitation [53]. Together, ammonia metabolism reflects an integrated process involving the intestine, liver, skeletal muscle, and kidneys, the dysregulation of which underlies hyperammonemia in cirrhosis **(**Fig. 2**)**.

### Role of inflammation and oxidative stress

Systemic inflammation and oxidative stress disrupt neuronal function and exacerbate cerebral vulnerability [25, 54]. Inflammatory cytokines contribute to brain endothelial cell activation and downregulation of tight junction proteins, such as zonula occludens-1 and claudin-5, resulting in blood–brain barrier dysfunction [55]. In patients with cirrhosis, serum inflammatory cytokines, including interleukin IL-6 and IL-18, are significantly elevated in those with CHE [56]. In addition, increased IL-6 is a risk factor for developing OHE in patients with cirrhosis [57]. Indeed, magnetic resonance imaging shows significant edema in those with CHE, and brain edema robustly correlates with serum IL-6 levels [58]. Importantly, lactulose treatment improved low-grade cerebral edema in patients with CHE [58]. In rats with cirrhosis, lipopolysaccharide administration precipitated pre-coma and worsened cytotoxic brain edema through the combined effects of hyperammonemia and inflammation. In a mouse model of acute liver failure, anti-tumor necrosis factor-α treatment significantly improved blood–brain barrier permeability, highlighting the critical role of inflammation and its potential as a therapeutic target [59].

Cerebral oxidative stress is frequently observed in patients with OHE, and hyperammonemia plays an important role in its development [60, 61]. In rats with chronic liver failure, brain edema occurred from the combined effects of hyperammonemia and systemic oxidative stress, with allopurinol, a xanthine oxidase inhibitor, treatment attenuating both oxidative stress and brain edema [62]. In rats with portosystemic shunts, induction of systemic oxidative stress in the setting of hyperammonemia led to brain edema, supporting that neither hyperammonemia nor oxidative stress alone, but their synergistic interaction, contributes to the pathogenesis of brain edema in HE [62]. Indeed, magnetic resonance findings of CHE showed a significant correlation with serum tumor necrosis factor (TNF) levels, whereas no such correlation was observed with serum ammonia levels [63]. Taken together, these findings suggest that, alongside hyperammonemia, systemic inflammation and oxidative stress are also critical contributors to blood–brain barrier dysfunction and brain edema in CHE.

### Role of gut-liver axis

**Fig. 3:**
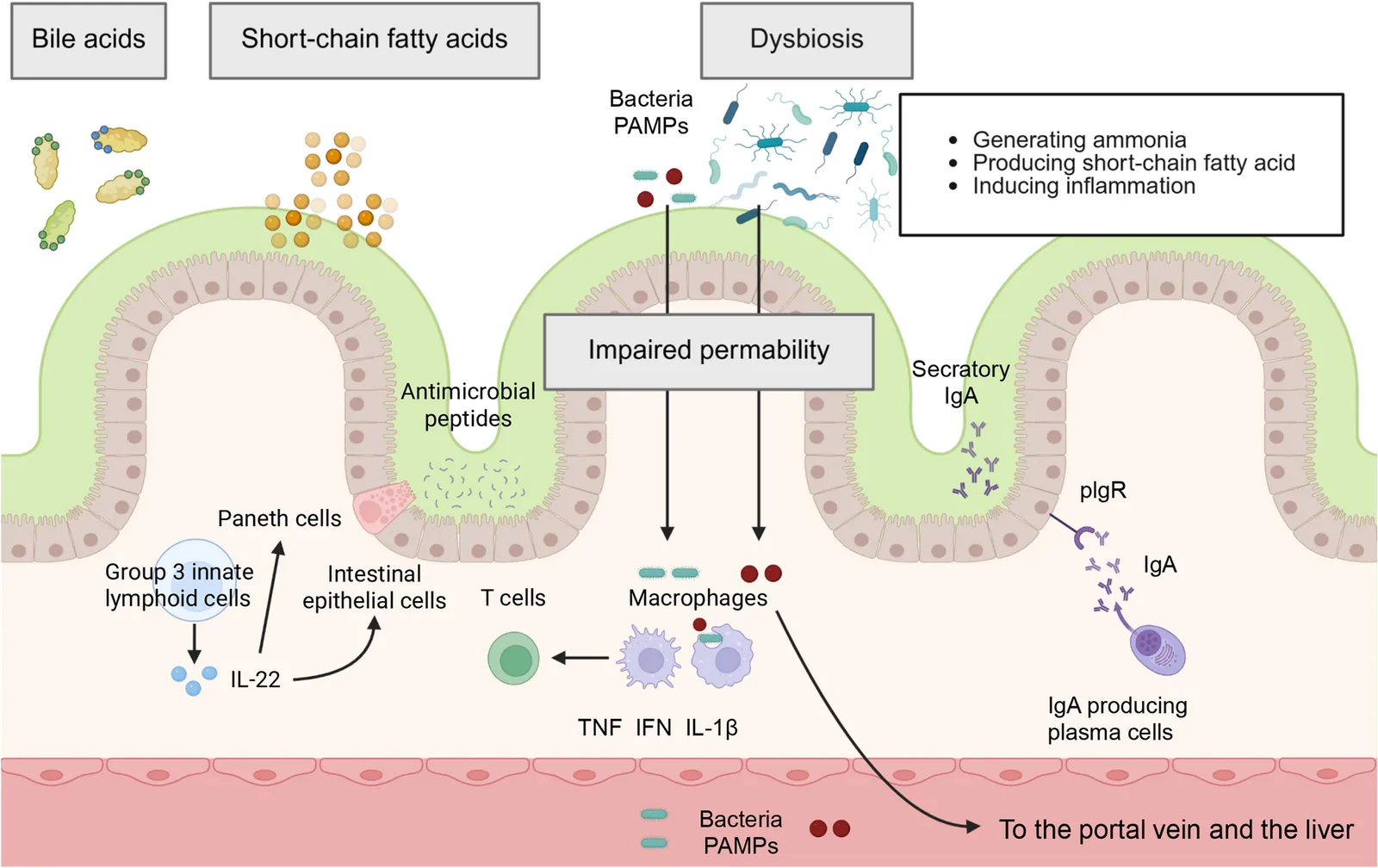
The gut-liver axis contributes to the pathogenesis of hepatic encephalopathy. Bacterial pathogen-associated molecular patterns (PAMPs) in the intestinal lumen are sensed by mucosal immune cells and initiate innate immune responses that shape subsequent adaptive immunity within the gut mucosa. Group 3 innate lymphoid cells produce interleukin-22 (IL-22), which acts on intestinal epithelial cells and Paneth cells to induce antimicrobial peptide production. Macrophages and T cells respond to microbial signals by producing inflammatory cytokines. In parallel, immunoglobulin A (IgA)-producing plasma cells generate IgA, which is secreted into the intestinal lumen as secretory IgA and contributes to immune exclusion of bacteria and microbial products. Gut microbiota–derived metabolites, including bile acids and short-chain fatty acids, further regulate intestinal barrier integrity and mucosal immune homeostasis by modulating epithelial cell function and immune cell activity. Disruption of these mucosal immune defenses and metabolite-mediated regulatory pathways facilitates the translocation of bacteria and PAMPs into the portal circulation, thereby delivering inflammatory signals to the liver and contributing to hepatic inflammation and hepatic encephalopathy. *PAMP* pathogen-associated molecular pattern, *IL-22* interleukin-22, *TNF* tumor necrosis factor, *IFN* interferon, *IL-1β* interleukin-1 beta, *IgA* immunoglobulin A, *pIgR* polymeric immunoglobulin receptor, *SCFA* short-chain fatty acid

Cirrhosis is associated with dramatic alterations of the gut microbiome [64], which likely contribute to CHE pathogenesis via disruption of the gut-liver-brain axis. Alterations in the gut–liver axis contribute to increased intestinal permeability, dysbiosis, and enhanced production of neurotoxic metabolites [10]. Short-chain fatty acids play a central role in maintaining gut homeostasis by serving as an energy source for intestinal epithelial cells, strengthening gut barrier integrity, and modulating immune and inflammatory responses [65]. In addition, secretory immunoglobulin A limits bacterial contact with epithelial cells and promotes adaptive immune responses via Peyer’s patches [66]. Dysregulated inflammatory mediators and microbial products, such as cytokines (e.g., TNF, IFN, IL-1β, IL-22) and endotoxins, are closely linked to barrier dysfunction and impaired intestinal homeostasis [10]. The maturation and function of barrier-associated immune cells (e.g., macrophages) are also shaped by the gut microbiome [67, 68]. Clinical evidence indicates that bacterial DNA translocation is strongly associated with OHE, highlighting gut permeability as a critical determinant in the pathogenesis of HE [69]. In CHE, the gut microbiota of patients show a significantly reduced Shannon diversity index compared with those without CHE, and specific gut microbial families have been linked to its presence, including *Lactobacillales*, *Enterobacteriaceae*, *Streptococcaceae*, *Lachnospiraceae*, *Clostridiales*, and *Bacteroidaceae* [70]. Another study found that the gut microbiota of patients with CHE were characterized by loss of *Eubacteriaceae* and *Sutterellaceae*, including *Eubacterium*, *Lachnospira*, *Parasutterella*, and *Erysipelotrichaceae*, and enrichment of *Clostridiales*, including *Odoribacter*, *Flavonifractor*, and *Coprobacillus* [71]. Collectively, these compositional shifts implicate multiple functional pathways relevant to CHE, including short-chain fatty acid production (e.g., *Lachnospiraceae*, *Eubacterium*) [72], inflammatory signaling and microbial product-related pathways (e.g., *Enterobacteriaceae*, *Streptococcaceae*) [73], and ammonia or nitrogen metabolism (e.g., *Clostridiales*) [74], rather than a single dominant mechanism. In addition, beyond lipid digestion, bile acids act as key signaling molecules that regulate hepatic metabolism, gut microbiota, intestinal integrity, and immune as well as glucose and lipid homeostasis through receptors such as the farnesoid X receptor and Takeda-G-protein-receptor-5 [75]. In fact, patients with CHE show altered bile acid profiles compared with those without, and such bile acids correlate with serum ammonia levels [76, 77]. Taken together, disruptions in short-chain fatty acid-related metabolism, gut-derived inflammation, bile acid signaling, and ammonia/nitrogen handling support a mechanistic contribution of the gut-liver axis to HE **(**Fig. 3**)**.

### Role of malnutrition and other systemic factors

Malnutrition is highly prevalent in patients with cirrhosis and represents a critical contributor to CHE [78]. Malnutrition leads to an imbalance in amino acids, characterized by decreased BCAAs and increased aromatic amino acids, resulting in reduced mammalian target of rapamycin signaling, impaired protein synthesis, hypoalbuminemia, and ultimately worsening of HE [41, 79, 80]. Furthermore, hypoalbuminemia reduces the binding capacity of circulating zinc, leading to zinc deficiency, which has been associated with the development of CHE and progression to OHE [20, 81]. Beyond its systemic metabolic effects, malnutrition also directly affects cerebral energy homeostasis. The brain has a high demand for continuous nutrient and energy supply, and malnutrition can induce cerebral energy failure [25]. Indeed, metabolomic profiling of cerebrospinal fluid samples from patients with HE has demonstrated impaired cerebral energy metabolism, supporting the presence of brain energy deficiency in this population [26]. In addition, fluid and electrolyte abnormalities further exacerbate vulnerability to CHE. Dehydration, often precipitated by diuretics or large-volume paracentesis, reduces effective renal perfusion and may impair renal ammonia excretion, leading to increased systemic ammonia levels. Furthermore, electrolyte imbalances, particularly hyponatremia and hypokalemia, further contribute to the development of HE. Hyponatremia reduces cerebrospinal fluid osmolarity, thereby impairing astrocyte volume regulation and increasing cerebral susceptibility to osmotic stress and ammonia accumulation, ultimately leading to astrocyte swelling [82]. Hypokalemia and acidosis increase renal ammonia production as part of acid–base compensation, thereby contributing to hyperammonemia and HE [83].

## Current testing for CHE

CHE is diagnosed using neuropsychological/neurophysiological tests. Because no single gold standard exists and agreement between tests is limited, reflecting assessment of different cognitive domains, test selection depends on locally validated norms and expertise [3, 4]. Standardized batteries such as the psychometric HE score (PHES) are considered reference tools, while simpler screening tests, including the Stroop test and animal naming test (ANT), are also available, each with distinct strengths and limitations **(**Table 2**)**. Accordingly, an understanding of the characteristics of each test is essential. We provide an overview of the major diagnostic modalities.

**Table 2:** Characteristics of covert hepatic encephalopathy testing

Test name	Duration	Strengths	Limitations	Key neurocognitive domains
NPT [21, 87]	15–20 min	Gold standard in Japan Consists of 4 paper-and-pencil tests Predicts progression to OHE	Time-consuming Requires specialized computer software Requires age adjusted scores	Attention, mental tracking, psychomotor speed, visuomotor/spatial function
PHES [84, 85]	15–20 min	International reference standard Consists of 5 paper-and-pencil tests Predicts progression to OHE	Time-consuming Requires age/sex-adjusted scores Repeated testing leads to learning effects	Attention, mental tracking, psychomotor speed, visuomotor/spatial function
Stroop test [90–93]	5 min	App-based Good diagnostic performance Predicts progression to OHE	Requires specialized computer software Affected by visual impairment Needs further local validation	Processing speed, inhibitory control
Quickstroop (Shortened) [96–100]	1 min	Extremely rapid App-based Good diagnostic performance Predicts progression to OHE	Requires specialized computer software Affected by visual impairment Needs further local validation	Processing speed (inhibitory control)
Animal naming test [104–110]	1 min	No specialized equipment Extremely rapid Predicts progression to OHE	Thresholds vary by culture (≤ 10–22) Needs further local validation	Semantic memory, executive function, processing speed
Critical flicker frequency [22, 89, 111, 112]	5 min	Independence from gender and education	Requires specialized equipment Affected by visual impairment	Cortical arousal and visual processing
Inhibitory control test [87, 113]	10 min	Moderate to high diagnostic accuracy	Requires specialized computer software Influenced by age/education Repeated testing leads to learning effects	Sustained attention, response inhibition

*NPT* neuropsychological test, *OHE* overt hepatic encephalopathy, *PHES* psychometric hepatic encephalopathy score

### Composite test batteries

The PHES is the most widely used reference test for the diagnosis of CHE. It is a composite battery consisting of five paper-and-pencil neuropsychological tests: the number connection test-A, number connection test-B, digit symbol test, line-tracing test, and serial dotting test [84, 85]. These tests assess attention, mental tracking, psychomotor speed, and visuomotor and visuospatial function, domains that are typically impaired in CHE. Performance on each test is expressed as an age- and sex-adjusted Z-score relative to healthy controls, and the overall PHES is calculated by summing the component scores. A PHES value of ≤ − 4 is the most commonly applied threshold in clinical practice and epidemiological studies to diagnose MHE, whereas more stringent cut-offs such as ≤ − 5 or ≤ − 6 have been used in some studies to define more severe impairment [84, 85]. Because completion of the full PHES can be time-consuming, the Working Group on HE recommends a simplified battery using two of the following tests—number connection test-A, number connection test-B, digit symbol test, and the block design test—with CHE defined by impairment in at least two tests [86]. Based on this statement, the Japanese Society of Hepatology recommends a computer-aided neuropsychological test (NPT), consisting of the number connection test-A, number connection test-B, digit symbol test, and block design test, as a gold standard test for CHE, with CHE diagnosed when at least two of these tests show abnormal performance [87]. The clinical utility of these tests is supported by evidence that CHE diagnosed using PHES or NPT is an independent predictor of OHE in patients with cirrhosis [21, 88]. However, these batteries typically require 15–20 min to complete, which limits their practicality in clinical settings [8]. Furthermore, repeated assessment can provide a learning effect which can reduce the diagnostic capacity in PHES [89].

### Stroop test and its shorter version

The Stroop test has emerged as a rapid point-of-care tool for CHE that can be completed within a short time using minimal equipment and operator burden [90–93]. The test comprises two task conditions that differ in cognitive demand: an off-state and an on-state, enabling the assessment of processing speed, cognitive control, and executive function. In the off-state, participants are required to identify the font color of a neutral symbol displayed on a digital screen. In contrast, the on-state introduces a strong conflict between word reading and color naming, requiring participants to suppress the meaning of the word and instead report the color of the font. The Stroop off-state primarily reflects psychomotor and processing speed, which are linked to subcortical arousal and basic cortical activation, whereas the on-state additionally probes cognitive flexibility and response inhibition, capturing the functional integrity of the anterior cingulate cortex and lateral prefrontal cortex [94, 95]. Previous studies have demonstrated that the Stroop performance shows high diagnostic accuracy and reproducibility for CHE and can also predict progression to OHE, with the full protocol typically requiring less than 10 min to complete [90–93]. To further streamline testing, a shortened version known as QuickStroop was recently developed and validated in the United States, which consists of two off-state runs and can be completed within one minute while preserving diagnostic accuracy comparable to the full version [96–99]. In Japanese patients, a shortened Stroop protocol, using two on-state runs administered in Japanese, has been shown to provide the highest diagnostic accuracy for detecting CHE [100]. This is not unexpected, as differences in language, cultural background, population norms, and patient characteristics may limit the direct applicability of QuickStroop to Japanese patients with cirrhosis. Moreover, despite its diagnostic performance, patient acceptability and real-world usability of Stroop-based testing remain important challenges. In fact, a study evaluating the feasibility of the Stroop test in patients with cirrhosis showed that 71% agreed to download the application, but actual use was recorded in only 32%, which may limit its practicality in clinical settings [101].

### Animal naming test

The ANT assesses cognitive function by measuring an individual’s ability to recall as many animal names as possible within a 60-s time limit [85]. The ANT mainly evaluates semantic memory, executive function, and processing speed, cognitive domains that are commonly impaired in patients with CHE/MHE [102, 103]. A major strength of the ANT is that it does not require any specialized equipment, allowing for quick and convenient bedside evaluation. Accordingly, the current guideline recommends the ANT as the only point-of-care diagnostic test, while highlighting the need for further validation [4]. The ANT is useful for the assessment of CHE/MHE, with reported areas under the curve ranging from 0.68 to 0.98, and for predicting OHE [104–110]. Validation studies have been conducted in many countries, with proposed cutoff values ranging from ≤ 10 to ≤ 22 [104–110]. Even within Europe, such as between Italy and Germany, the sensitivity and specificity differ when the same cutoff is applied [104, 105]. These discrepancies are presumed to reflect differences in cultural background, educational systems, underlying liver disease characteristics, diagnostic methods, the definitions of CHE and MHE, and the approaches used to determine cutoff values. In addition, although the widely used version of the ANT requires adjustment for age and educational attainment, a study from Japan has reported that such adjustments did not substantially change the diagnostic accuracy [109]. Given that the requirement for age- and education-based correction substantially limits its applicability, addressing this issue is essential for the broader adoption of the ANT.

### Other tests for CHE

Critical flicker frequency and the inhibitory control test are computerized point-of-care tools used for the assessment of CHE/MHE, each targeting distinct neurofunctional domains. Critical flicker frequency exploits the phenomenon that flickering light is perceived as continuous above a threshold frequency, with patients with CHE exhibiting lower flicker perception thresholds than those without [111]. Although critical flicker frequency is easy to perform, the proportion of patients identified as abnormal by critical flicker frequency often differs from that detected by other diagnostic tests, raising concerns regarding its diagnostic accuracy [22, 112]. Although it is not affected by gender and education, critical flicker frequency is limited by visual impairment, color blindness, retinopathy, and test-quality issues [89].

The inhibitory control test assesses attention and response inhibition in which patients view a rapid sequence of letters displayed on a screen and are instructed to press a response key only when the letter “X” is immediately followed by “Y” (target), while withholding responses to all other letter combinations (lures) [113]. The inhibitory control test demonstrates moderate to high diagnostic accuracy for CHE and has also been shown to predict progression to OHE [89]. However, it can be influenced by age and education, and repeated administration can lead to practice-related improvements that complicate the assessment [89].

## Current treatment for CHE

Since CHE is a well-established risk factor for progression to OHE, therapeutic intervention is generally recommended once CHE is diagnosed. However, no randomized controlled trials have rigorously evaluated whether treatment of CHE reduces the risk of OHE or mortality [4]. Therefore, current guidelines vary in their recommendations on whether CHE warrants pharmacologic treatment, and consensus is lacking regarding the timing of initiation, treatment duration, and discontinuation criteria. The American Association for the Study of Liver Diseases (AASLD) guidelines do not recommend routine treatment of MHE/CHE and suggest consideration on a case-by-case basis, particularly in patients with CHE or West Haven grade I HE [2]. The recent European Association for the Study of the Liver (EASL) guidelines support treatment with non-absorbable disaccharides (and/or rifaximin) in patients with CHE, based on accumulating evidence of its clinical impact [4]. In the LiveSMART randomized controlled trial, lactulose and Tai-Chi significantly reduced falls in patients with cirrhosis and portal hypertension without prior OHE [114]. A recent randomized trial of rifaximin solid soluble dispersion aimed at preventing a first episode of OHE in patients with portal hypertension did not fulfill the primary endpoint [115]. Although neither of these trials specifically targeted CHE nor demonstrated a significant reduction in OHE, many of the enrolled patients were likely to have CHE, and the results of the LiveSMART trial suggest potential clinical benefits of CHE-directed therapy beyond OHE prevention. CHE often presents with nonspecific symptoms, and differentiation from other conditions can be challenging, resulting in uncertainty regarding treatment indication in some cases [121]. Therefore, the EASL guideline proposes the initiation of therapy in diagnostically challenging cases and the use of treatment response as part of the diagnostic process [8]. Given this background, a comprehensive assessment of liver functional reserve, quality-of-life impairment, sleep disturbances, fall risk, and driving habits is essential for both determining treatment initiation and evaluating therapeutic response.

### Nutritional optimization and BCAA supplementation

Because energy deficiency and amino acid imbalance are closely involved in the pathophysiology of CHE, nutritional optimization represents the first step of management. Current recommendations generally target an energy intake of approximately 35 kcal/kg/day, with a protein intake of 1.2–1.5 g/kg/day to prevent catabolism and preserve muscle mass [116, 117]. In a randomized controlled trial involving patients with cirrhosis and CHE, dietary intervention with protein intake of 1.0–1.5 g/kg/day led to significant improvement in CHE and was associated with more favorable clinical outcomes compared with a standard diet [118]. Although protein loading has historically been thought to precipitate hyperammonemia and worsen HE, such a condition appears to be uncommon in real-world clinical practice [116, 117]. Importantly, protein restriction may exacerbate catabolic states and contribute to sarcopenia, highlighting the need to maintain adequate protein intake. BCAA supplementation corrects amino acid imbalance and is effective for HE in meta-analyses; however, its efficacy in CHE warrants further investigation [119]. Regarding protein sources, randomized controlled trials have shown that animal-based proteins may lead to greater increases in ammonia levels compared with plant-based proteins, suggesting that protein quality, in addition to quantity, may influence CHE pathophysiology [120]. A randomized cross‑over trial in patients with cirrhosis and chronic encephalopathy further demonstrated that a predominantly vegetable‑protein diet improved nitrogen balance, lowered ammonia and related metabolic markers, and modestly improved encephalopathy compared with an isocaloric, isonitrogenous animal‑protein diet [121]. Further studies are warranted to clarify the optimal composition of dietary protein in this population.

### Lactulose

Lactulose, a non-absorbable synthetic disaccharide, is metabolized by colonic bacteria into short-chain organic acids, such as acetic and lactic acid, leading to acidification of the intestinal lumen and suppression of ammonia-producing bacteria [122]. In addition, lactulose converts freely diffusible ammonia into non-absorbable ammonium ions, thereby reducing intestinal ammonia absorption, while its cathartic effect enhances fecal nitrogen excretion and lowers systemic ammonia levels [122]. Lactulose is the most extensively studied pharmacological therapy for CHE. In a network meta-analysis, lactulose was associated with significant improvement in CHE as well as a marked reduction in the risk of progression to OHE [123]. Indeed, a recent study demonstrated that lactulose is the most frequently used therapeutic option for CHE [8].

### Rifaximin

Rifaximin, a non-absorbable gut-selective antibiotic, modulates intestinal enzymatic activity, thereby suppressing inflammatory cytokine production, improving gut barrier function, and maintaining intestinal homeostasis [124, 125]. In a randomized controlled trial, rifaximin attenuates gut-derived systemic inflammation by suppressing oralization-associated, mucin-degrading sialidase-rich species (e.g., *Streptococcus* and *Veillonella*) and promoting a barrier-repairing intestinal environment [126]. In addition, rifaximin reduces ammonia production by decreasing small intestinal glutaminase activity and increasing intraluminal glutamine content [124]. Meta-analyses comparing rifaximin with placebo or no intervention consistently demonstrate an overall beneficial effect on HE [127]. Notably, this effect appears to be more pronounced in patients with CHE, as well as in studies evaluating the prevention of OHE [127]. Another network meta-analysis indicated that rifaximin is associated with significant improvement in CHE, supporting the role of rifaximin as an effective therapeutic option in the early stages of the disease [123]. Accordingly, rifaximin is recognized as the second most commonly used therapeutic agent for the treatment of CHE, following lactulose [8].

### Other treatments

Regarding other treatments, a randomized controlled trial demonstrated that intravenous albumin infusion reversed MHE in patients with cirrhosis [128]. Several other agents, including probiotics, L-ornithine L-aspartate, zinc supplementation, and levocarnitine, have been suggested to be beneficial for the treatment of CHE [129–131]. However, the available evidence remains limited due to a lack of adequately powered randomized controlled trials specifically targeting CHE, heterogeneity in study designs, and regional differences in the formulations used. Given that multiple therapeutic options are available for CHE, treatment strategies should be individualized by considering not only efficacy and adverse effects but also the patient’s disease severity, lifestyle, preferences, and economic burden.

## Challenges in CHE

### Implementation for CHE testing and multidisciplinary team

One of the greatest challenges in the clinical management of CHE is the low rate of screening implementation. Although screening for CHE is recommended for all patients with cirrhosis, testing is not performed in a substantial proportion of patients in real-world practice. Indeed, survey data indicate that more than one-third of physicians involved in cirrhosis care do not perform any CHE testing at all [7, 8]. Physician survey studies have identified lack of time, financial burden, shortage of examiners, and the absence of a standard test as major barriers to testing for CHE [7, 8]. Given this background, point-of-care tests, such as the Stroop test and the ANT have attracted attention as practical options; however, challenges remain, including limitations related to testing platforms, accessibility, and the strength of supporting evidence. To establish a sustainable care system for CHE, diagnostic tests should be brief, equipment-free, and easily administered by any healthcare professional, thereby enabling multidisciplinary collaboration and the development of a seamless algorithm from diagnosis to treatment.

A key strategy to improve CHE detection and management is the implementation of a multidisciplinary team approach [8]. Liver functional reserve, nutritional status, sarcopenia, medication use, and CHE are robust determinants of outcomes in cirrhosis, highlighting the need for comprehensive and coordinated care. Within this framework, nurses can monitor cognitive changes and support treatment adherence, dietitians can incorporate CHE screening into nutritional assessment and counseling, physical therapists can address sarcopenia and frailty, and pharmacists can optimize medication regimens and avoid potentially inappropriate therapies. Such multidisciplinary collaboration may reduce barriers to screening, promote earlier detection of CHE, and enable more effective and sustainable integration of CHE management into cirrhosis care [8]. Supporting this multidisciplinary approach, a specialized HE testing clinic integrating structured cognitive testing with medication review and targeted referrals showed that many cognitive complaints were not due to CHE and that test-guided counseling and shared decision-making enabled more rational, coordinated use of HE therapy [132].

### Biomarkers and risk stratification

Although current guidelines recommend universal testing for CHE in patients with cirrhosis, the use of biomarkers may help reduce the overall burden of CHE testing. However, there is currently no validated blood-based or imaging biomarker that can reliably diagnose CHE. Although serum ammonia levels are robustly related to the presence of CHE and development of OHE, their positive predictive value is low [3, 21]; therefore, they may be useful for excluding CHE, but definitive diagnosis requires formal CHE testing. Similarly, inflammatory markers such as interleukin-6 have been associated with CHE but also lack specificity [133]. Some reports have shown serum albumin levels as a biomarker for CHE [21, 134], and others suggested several candidate markers including vitamin D levels and neuroinflammation- and neuroinjury-related markers such as S100B, neurofilament light chain, and glial fibrillary acidic protein [135–138].

Since risk stratification from biochemical parameters alone is limited, the use of other clinical variables has gained attention. Given that sarcopenia is a key component of CHE pathophysiology, it is reasonable to test for CHE in patients with reduced muscle mass or muscle strength [44–46]. Speech patterns also represent a potential tool for identifying patients with CHE [139]. Multidimensional scoring systems may help identify patients with CHE or progression to OHE. A simplified CHE screening score was developed using age, sex, and selected items from the Sickness Impact Profile, a validated quality-of-life questionnaire [140]. Furthermore, a highly accurate clinical score was developed with a combination of ascites, history of OHE, serum albumin levels, quality-of-life assessment, and the ANT [141]. Despite good discriminative performance, the complexity of these models may limit their practicality in clinical practice. To address this gap, the simple CHE score has been proposed, incorporating only two variables: hypoalbuminemia (albumin ≤ 3.5 g/dL) and hyperammonemia (ammonia ≥ 80 μg/dL) [21]. Used as the first step in a two-step approach, formal CHE testing is subsequently performed only in patients who screen positive, allowing further risk stratification while substantially reducing the burden of CHE testing in clinical practice [21].

### Geriatric cirrhosis

**Fig. 4:**
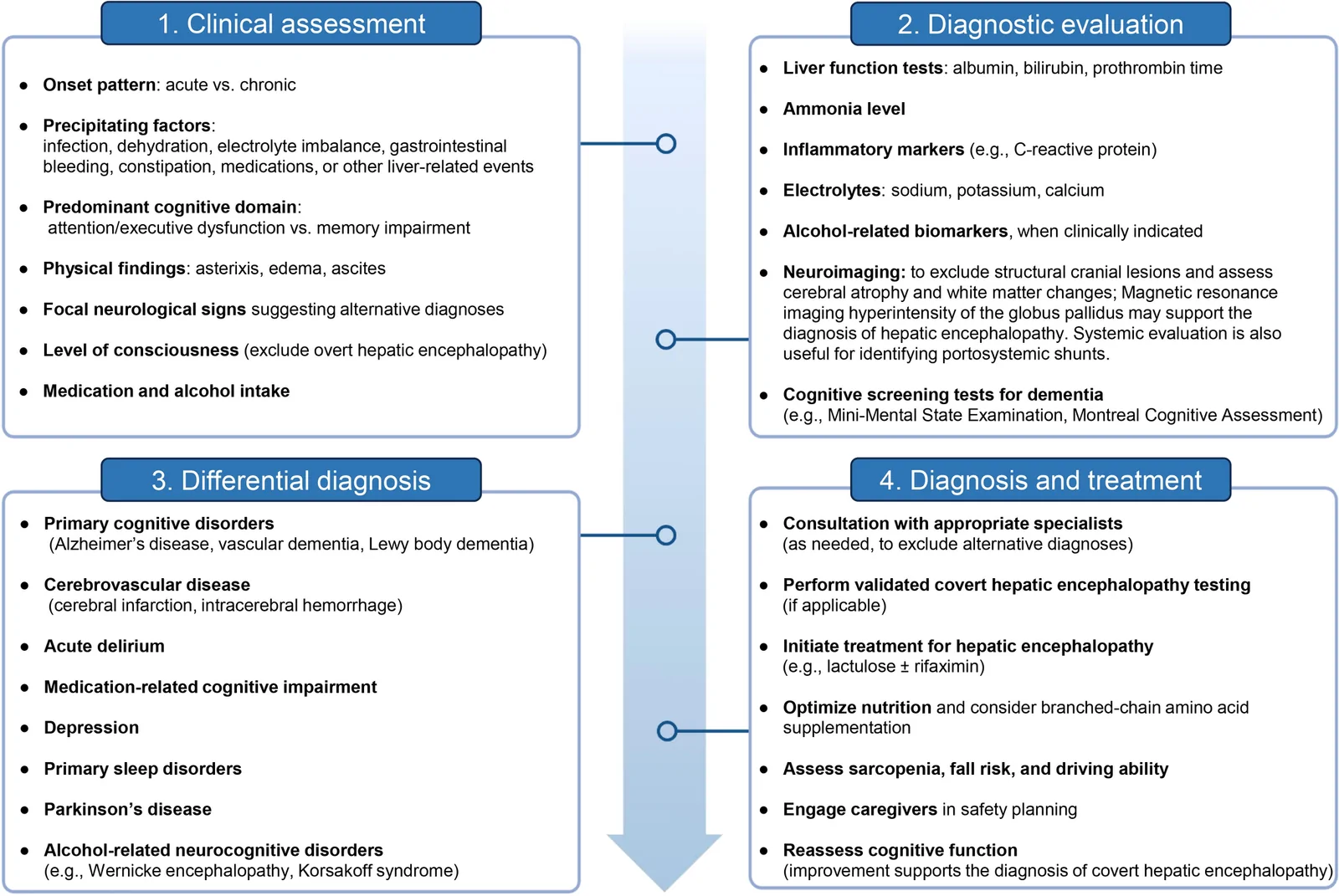
Clinical approach for evaluation and management of covert hepatic encephalopathy in geriatric cirrhosis. Initial evaluation should include assessment of the onset pattern, comorbidities, medication use, and alcohol intake. The predominant cognitive domain should be characterized, along with physical findings. Focal neurological signs should be assessed to exclude alternative structural diagnoses. Diagnostic evaluation includes laboratory data and medical imaging. Differential diagnosis includes delirium, dementia, cerebrovascular disease, medication-related cognitive impairment, depression, sleep disorders, Parkinson’s disease, and alcohol-related neurocognitive disorders. After the exclusion of alternative causes, validated covert hepatic encephalopathy testing is recommended if applicable. Management includes lactulose with or without rifaximin, nutritional optimization with consideration of branched-chain amino acid supplementation, and engagement in safety planning, including the assessment of sarcopenia, fall risk, and driving ability. Reassessment of cognitive function helps confirm the diagnosis of covert hepatic encephalopathy

Aging of the cirrhosis population presents additional challenges in the diagnosis and management of CHE. Older patients frequently exhibit age-related cognitive decline, sarcopenia and frailty, sensory impairment, and comorbid neurodegenerative conditions, all of which complicate the interpretation of CHE testing. CHE must be differentiated from other conditions, including dementia, cerebrovascular diseases, acute delirium, depression, sleep disorders, Parkinson’s disease, alcohol-related neurocognitive disorders, and medication-related cognitive impairment. In particular, distinguishing CHE from mild cognitive impairment or early dementia remains a major clinical challenge. In fact, a recent study found that HE, but not other decompensating events such as ascites, was independently associated with a diagnosis of dementia [142]. Most CHE studies have systematically excluded very elderly patients, and validated cutoff values for CHE testing in geriatric cirrhosis have not been established. A structured diagnostic approach, including clinical evaluation, differential diagnosis, and assessment of therapeutic response when appropriate, is essential to avoid misdiagnosis in elderly patients with cirrhosis, as the response to HE treatment may help confirm the diagnosis (Fig. 4) [4, 143]. Because prevention of OHE is a key clinical goal, close monitoring based on established OHE risk markers such as hypoalbuminemia and hyperammonemia represents a practical approach [144]. Nevertheless, significant challenges remain in the early diagnosis of CHE in elderly patients and in demonstrating that treatment can effectively reduce risks related to quality-of-life impairment, driving performance, and falls.

### Driving and falls

CHE is well known to increase the risk of falls and fall-related fractures, as well as to impair driving performance, thereby contributing to traffic accidents [14–19]. Despite this, standardized methods to assess these risks and effective preventive strategies remain inadequately studied. The LiveSMART trial demonstrated that lactulose combined with Tai-Chi reduced falls and fall-related injuries in patients with cirrhosis without prior OHE [114]. These findings suggest that CHE may warrant consideration as a therapeutic target from a fall-prevention perspective, while highlighting the need to define how fall risk should be systematically monitored and managed. Similar challenges exist for driving impairment and traffic safety. Although tools, such as computerized in-office assessments with on-road evaluation and virtual reality-based driving tests have been developed, practical and easily deployable screening methods are still lacking [19, 145]. Moreover, clinical trials using traffic violations or accidents as outcomes face major ethical and methodological barriers, including safety concerns and large sample size requirements. Finally, even when CHE is identified as conferring a high risk for traffic accidents and therapeutic intervention is initiated, determining how best to address patients’ actual driving practices remains clinically and ethically complex, particularly in societies with limited alternative transportation options.

### New treatments for HE

In addition to established therapies, several emerging approaches are being explored for the treatment of HE. Regarding dietary interventions, a pilot open-label trial of resistant potato starch in patients with cirrhosis and previous OHE is currently underway in the United States (NCT06425380) [146]. Polyethylene glycol, an effective osmotic laxative, has been shown to have potential in improving CHE [147]. Nitrogen scavengers, particularly glycerol phenylbutyrate, represent a rational strategy for secondary prevention, as they lower ammonia through enhanced urinary nitrogen excretion [148]. L-ornithine phenylacetate reduces ammonia via a renal nitrogen disposal mechanism, and in a randomized controlled trial, shortened the median time to clinical response compared with placebo, although this difference did not reach statistical significance [149]. Gut-directed therapies targeting intestinal inflammation and bacterial translocation are also under investigation. Yaq-001 is a gut-restricted, non-absorbable carbon-based adsorbent developed to sequester endotoxin and other gut-derived toxins without disrupting the gut microbiome, thereby attenuating bacterial translocation-driven systemic inflammation, providing a rationale for its potential application in HE [150]. Microbiome-based interventions, including fecal microbiota transplantation and defined live biotherapeutics, such as VE303, directly target the gut–liver–brain axis and may represent the next generation of HE treatment strategies. In the phase II double-blind, placebo-controlled THEMATIC trial, fecal microbiota transplantation was safe in patients with cirrhosis and recurrent HE, with no treatment-related adverse events [151]. Importantly, the proportion of liver-related hospitalizations or HE recurrences was lower among patients receiving any fecal microbiota transplantation compared with the placebo group [151]. VE303 is an oral, defined consortium of eight clonal bacterial strains intended to modulate the gut–liver–brain axis by promoting strain engraftment and beneficial metabolites such as butyrate [152]. In a randomized placebo-controlled phase IIA trial of patients with prior OHE, VE303 was well tolerated and showed a trend toward improved PHES [152]. Finally, reformulations of existing agents, such as rifaximin soluble solid dispersion tablets, may improve pharmacokinetic or practical aspects of therapy [153].

## Conclusions

In conclusion, evidence on CHE has advanced substantially over the past two decades, highlighting its growing importance in cirrhosis care. Despite this, CHE remains underdiagnosed in many patients because testing is time- and resource-intensive and because optimal strategies for risk stratification are still evolving. Future progress will require strengthening the evidence base and standardizing point-of-care tests, establishing validated biomarkers and risk stratification frameworks to define who should be screened and treated, and addressing the unique diagnostic challenges in geriatric cirrhosis. In parallel, continued development of effective and implementable therapies will be essential to clarify the clinical value of early CHE detection and intervention. Ultimately, accelerating the real-world implementation of CHE care will depend on comprehensive, multidisciplinary collaboration that links standardized testing, risk-based pathways, and feasible treatment strategies to improve patients’ quality-of-life and outcomes.
